# Carbon and oxygen isotope characteristics of carbonate rocks in the Mesoproterozoic Jixian System of the Ordos Basin and their implications

**DOI:** 10.1038/s41598-023-41297-w

**Published:** 2023-08-28

**Authors:** Liu Xiaofeng, Hong Zenglin, Liang Jiwei, Guo Xiaodan, Xue Xuping, Li Shifeng

**Affiliations:** 1 School of Land Engineering, Chang’an University, Xi’an, 710054 People’s Republic of China; 2 Shaanxi Institute of Geological Survey, Xi’an, 710054 People’s Republic of China; 3 School of Earth Science and Resources, Chang’an University, Xi’an, 710054 People’s Republic of China; 4 Shaanxi Provincial Mineral Geological Survey Center, Xi’an, 710000 People’s Republic of China

**Keywords:** Geochemistry, Geology

## Abstract

The paleoenvironment of Jixian carbonate rocks in the Mesoproterozoic Ordos Basin is studied by carbon and oxygen isotope analyses, diagenetic environment analysis, and the restoration of paleosalinity and paleotemperature. The results indicate that the carbonate rocks of the Jixian System have always been in a near-surface environment and have not been deeply buried. The ranges of variation in δ^13^C_PDB_ and δ^18^O_PDB_ are relatively narrow, ranging from − 5.75 to 1.41‰ and − 8.88 to − 4.01‰, respectively, which is consistent with the stable tidal flat sedimentary environment during the Mesoproterozoic in the study area. The paleosalinity (Z) values range from 111.7 to 127.1, and the paleotemperature (T) values range from 32.7 to 57.33 °C, indicating a relatively warm paleoclimatic environment during the Mesoproterozoic era in the study area. The analysis shows that in a warm paleoclimatic environment, although carbon and oxygen isotopes, Z, and T have certain fluctuations, their ranges are relatively small, reflecting to some extent the stable tectonic environment of the study area during the Mesoproterozoic era. Comprehensive research shows that the Ordos Basin had a warm climate and a stable tectonic environment in the Mesoproterozoic, which may be a good response to the North China Block's position near the equator and continuous thermal subsidence in the Mesoproterozoic.

## Introduction

The Mesoproterozoic is an important stage in the geological history of supercontinent convergence and fragmentation, during which a global tectonic event occurred, and the analytical method represented by carbon and oxygen isotopes gives a more reasonable analysis^1–5^. At the end of the twentieth century, carbon and oxygen isotope methods were widely used in studying the aggregation and fragmentation of multiple supercontinents and Snowball Earth in the Proterozoic, with satisfactory results^6–10^. Carbonate rocks contain much information about the characteristics of sedimentary environments, among which oxygen isotopes can determine the temperature and salinity of ancient seawater^11^; the carbon isotope composition records the interaction between the global carbon cycle and the atmosphere–ocean–biosphere^12^, and it can be widely used for global-scale or regional stratigraphic correlation^13^; at the same time, carbon isotopes can reflect ocean cycles, ocean productivity, the supply of terrestrial debris, etc., providing the possibility for studying the ancient marine environment. Until now, carbon and oxygen isotopes of carbonate rocks have been widely used for global stratigraphic division and correlation, as well as paleotemperature, paleoenvironment, and paleoclimate reconstructions.

Scholars have conducted extensive research on the use of carbon and oxygen isotopes in Proterozoic carbonate rocks. Li et al.^14^, through isotope analysis of Proterozoic organic matter and paragenetic carbonate rocks in the Yanshan Basin, suggested that carbon isotopes can be a good record of biological community changes with the rise and fall of seawater. Chu et al.^15^ conducted a systematic analysis of the carbon isotope characteristics of the Proterozoic carbonate rocks in the Jixian System and proposed that the high carbon isotope values during two time periods on the profile may be responses to two tectonic events globally. Luo et al.^16^ studied the Mesoproterozoic strata in the Kuancheng area and showed that carbon and oxygen isotopes are closely related to algal blooms and sea level fluctuations. The successful application of carbon and oxygen isotope methods in Proterozoic and other ancient strata and the analysis of marine carbonate rocks at different ages by carbon and oxygen isotope analytical methods show that they can be an effective means to restore the ancient environment^17–21^.

Due to regional and developmental differences, research on the carbonate rocks in the Ordos Basin mainly focuses on Ordovician strata^22–24^. With the continuous intensifying of research, the Mesoproterozoic carbonate rocks in the southern part of the basin have also entered people's areas of focus^25,26^. The Mesoproterozoic Jixian System strata are widely distributed in the southwest margin of the Ordos Basin, and a large number of carbonate rocks are present, which provides good conditions for studying the paleoclimatic environment of the Mesoproterozoic Jixian System. Stable isotopes preserved in marine carbonate rocks can effectively represent the original information of sedimentary environments. By collecting Jixian System carbonate rocks and analyzing their trace elements and carbon and oxygen isotopes, this study can compensate for the lack of Mesoproterozoic geochemical data in this area to a certain extent, which is of great significance to studying the characteristics of the paleoclimate and environment in the southwest margin of the Ordos Basin.

## Geological setting

**Figure 1:**
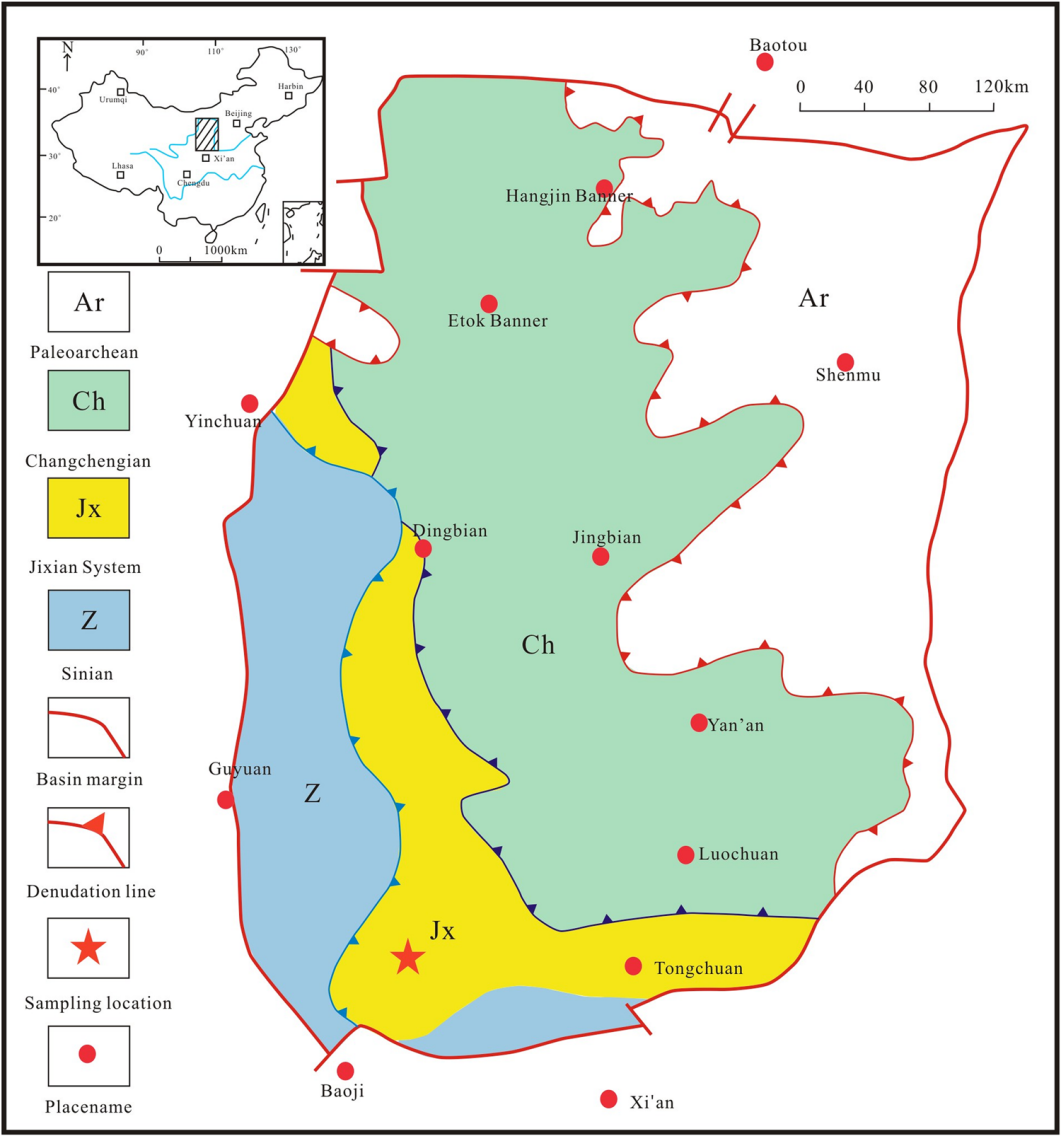
Map of the precambrian paleogeology in the Ordos Basin^33,34^.

During the Mesoproterozoic, the Helan and Qinjin aulacogen troughs were developed in the southwestern part of the North China Block on the basis of the Qin Qi continental rift^27^. The Ordos Block is influenced by the continuous rifts and depressions in the Qinjin aulacogen and the interior of the block, resulting in a continuous deepening of the water body (with a relatively small amplitude)^28–30^. In this context, a set of thick Mesoproterozoic Jixian System carbonate rocks was deposited in the southwestern margin of the Ordos Basin, with a gradually increasing sedimentary thickness from the margin of the block (greater than 225 m) to the interior of the block (greater than 616 m)^31,32^. The Jixian System is an uplifted ancient land in the northeastern part of the Ordos Block and a sedimentary depression area in the southwest margin. Shallow water shelf clastic and carbonate rock deposits developed, forming the Jixian System; these rocks are represented by the Wangquankou Group in the western margin of the basin and the Luonan Group in the southern margin; they are mainly distributed in the area bordering Shaanxi Gansu and northern Ningxia (Fig. 1^33^), which is located approximately in the middle section of the southwest margin of the North China Block^34^.

**Figure 2:**
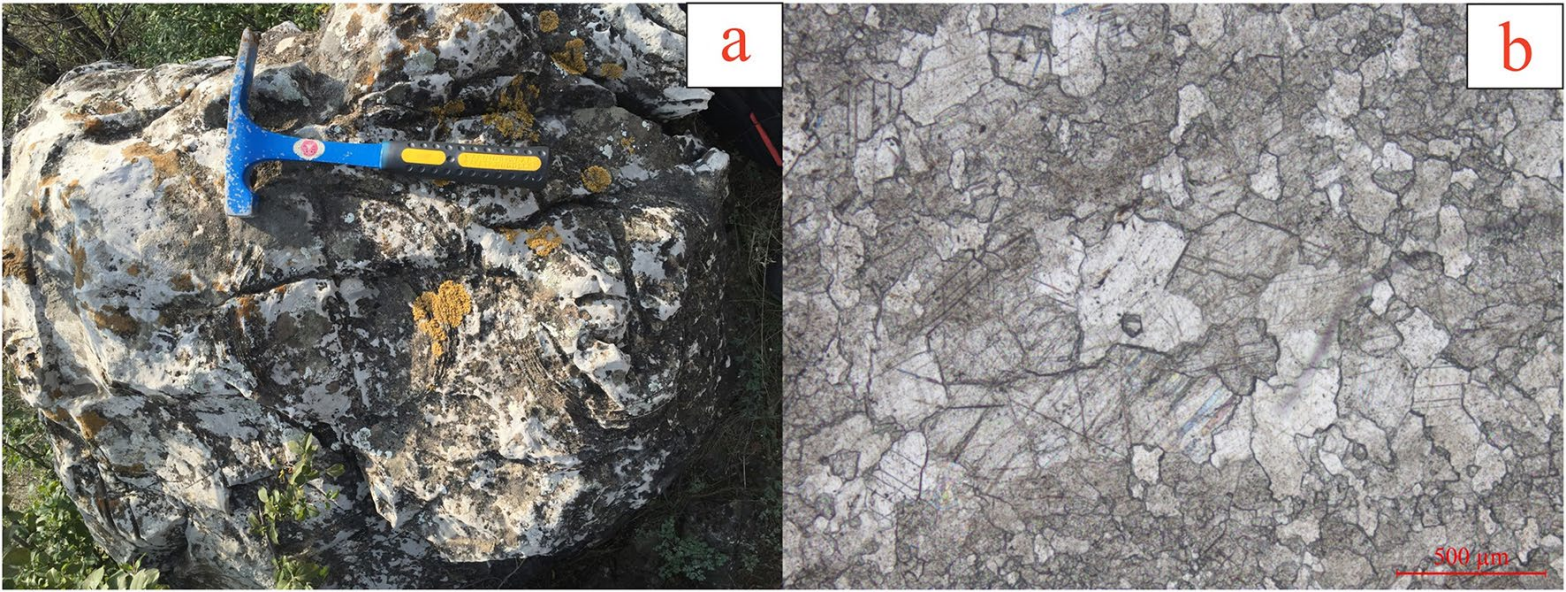
Stratigraphic and microscopic characteristics of mesoproterozoic carbonate rocks (**a** carbonate rocks containing algal laminate; **b** crystalline dolomite).

The study area is located near Qishan County in the southwestern margin of the Ordos Basin. The maximum exposed thickness of the Mesoproterozoic carbonate rocks in this area exceeds 1350 m. The lithology is mainly tidal flat siliceous banded algal laminated dolomite (Fig. 2), which contains abundant stromatolites. It is a set of well-preserved and basically unmodified sedimentary rock series. This depositional period was the peak of stromatolite development^33,35^.

## Sampling and analytical methods

### Sample description

The experimental samples were collected from the Mesoproterozoic carbonate rock field outcrop in the southwest margin of the Ordos Basin. During sampling, samples with fresh sections were selected, and parts such as those affected by later weathering and calcite veins were avoided as much as possible to ensure the accuracy of the test results. A total of 46 thin section samples and 30 samples for carbon and oxygen isotope analyses were collected for thin section microscopic identification and carbon and oxygen isotope analyses, respectively. Carbonate rocks have a good degree of recrystallization, a good crystal shape and large grains, which range from powder crystals to coarse crystals. Powder crystalline and fine crystalline dolomite are relatively dense, partially contain algae or mud, are mostly semi-self- to self-shaped, and have a visible "foggy core–bright edge" structure; the fine to medium crystalline dolomite has crystals that are mostly heteromorphic and in contact with each other in a planar or inlaid manner; the medium to coarse crystalline dolomite has relatively straight crystal edges, and the crystals are mostly self-shaped.

### Carbon and oxygen isotope analyses

The carbon and oxygen isotope samples that were collected for this study were all dolomite, and the sampling horizon was the Mesoproterozoic Jixian System. The samples were pretreated in the Open Research Laboratory of Mineralization and Kinetics, Ministry of Land and Resources, Chang'an University. The samples were crushed to 200 mesh (0.074 mm) with the JC6 bench-type jaw crusher and vibrating disk crusher of Beijing Greiman Instrument Equipment Co., Ltd. for subsequent geochemical testing. The testing and analysis of trace and major elements were completed by the Open Research Laboratory of Mineralization and Dynamics of the Ministry of Land and Resources of Chang'an University. A total of 0.1000 ± 0.0002 g of sample was weighed and placed in a 30 ml polytetrafluoroethylene crucible. Then, 10 ml of HNO_3_ + HCIO_4_ + HF (2:2:1) mixed acid was added, it was covered, and the sample was dissolved on a temperature-controlled electric heating plate. After smoking, the samples were kept at 100 °C for 3 h. After drying, 5 ml of aqua regia (1:1) was added and extracted while hot. After cooling, the mixture was transferred to a 50 ml volumetric flask, diluted with distilled water, shaken well, and set aside. Major elements were analyzed using ICP‒OES, while trace elements were analyzed using ICP‒MS. Carbon and oxygen isotopes were determined by Beijing Kehui Testing Technology Co., Ltd. Then, 0.5 mg of the sample was weighed and placed into the Gas-Bench automatic sampling system, high-purity He gas was added for cleaning, and it was dissolved at a constant temperature of 70 °C for 2 h at 100% H_3_PO_4_. The testing instrument was a Thermo Fisher Scientific MAT235 isotope mass spectrometer; Vienna Pee Dee Belemnite (VPDB) was used as the standard sample, and the testing error was ± 0.02 ‰.

## Results

### Data reliability

**Figure 3:**
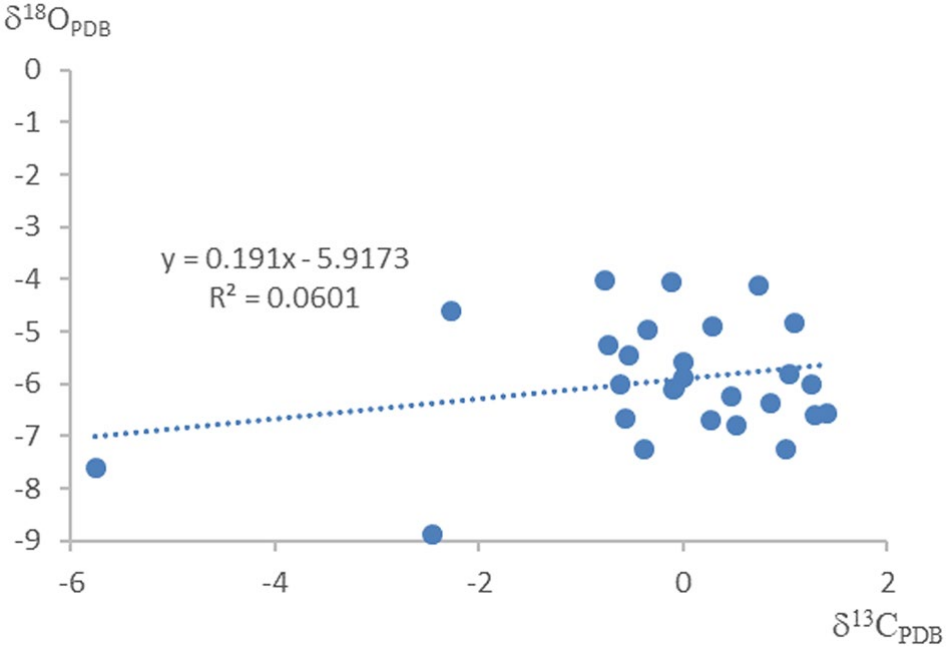
δ^13^C–δ^18^O relationship of middle proterozoic carbonate rocks in the Ordos Basin.

The ancient marine carbonate rocks are prone to alteration during later diagenesis and lose their original sedimentary information, so an initial assessment is required before studying the carbon and oxygen isotopes^36,37^. The samples collected in this study are all micritic dolomite, with a particle size of less than 5 μm, and their degree of modification during diagenesis is relatively weak^38^. Research has shown that after sedimentation, especially under the influence of atmospheric water circulation, carbonate rocks experience the loss of Sr and the addition of Mn^39^. Therefore, the Mn/Sr ratio can be used to determine whether the carbon isotope composition has undergone changes. Kaufman et al.^40^ proposed that carbonate rocks with Mn/Sr < 10 can usually retain their original carbon isotope composition. The analytical results in Table 1 reveal that the Mn/Sr values in the samples are all less than 10, indicating that the original carbon isotope composition of the samples has been preserved. Oxygen isotopes are sensitive to changes in the postdiagenetic environment. It is generally believed that diagenesis has a great impact on rocks when δ^18^O_PDB_ < − 10‰, and carbon and oxygen isotopes change strongly; when δ^18^O_PDB_ < − 5‰, the rock is affected by diagenesis, but the carbon and oxygen isotope composition and content change slightly^40,41^. The test data reveal that except for the KH-05 sample, all other samples are greater than − 10‰, indicating that the data are generally available. To ensure the reliability of the analysis, KH-05 and KH-09 samples were removed from the subsequent analysis. At the same time, many scholars use the lack of correlation between carbon and oxygen isotopes as a basis to determine the originality of carbon and oxygen isotopes in rocks^42,43^. The correlation between carbon and oxygen isotope contents in this study is poor (the fitting equation is y = 0.191x − 5.9173, and the correlation coefficient is 0.0601), showing a discrete feature overall (Fig. 3). Therefore, the samples used in this study are less affected by diagenesis and basically maintain the characteristics of the original sediment, which can meet the requirements of paleoenvironmental analysis.

**Table 1:** Geochemical data of mesoproterozoic carbonate rocks in the Ordos Basin.

Sample no	δ^13^C_V-PDB_ ‰	δ^18^O_V-PDB_ ‰	Mn (ppm)	Sr (ppm)	Mn/Sr	Z value	T/℃
KH-01	− 0.77	− 4.01	29.70	217.16	0.14	123.7	32.71
KH-02	− 0.73	− 5.27	28.60	39.38	0.73	123.2	38.89
KH-03	− 0.61	− 6.02	36.73	55.17	0.67	123.1	42.62
KH-04	0.47	− 6.23	22.94	32.86	0.70	125.2	43.71
KH-05	− 9.83	− 15.79	188.77	23.90	7.90		
KH-06	0.52	− 6.81	59.44	15.93	3.73	125.0	46.60
KH-07	1.41	− 6.56	42.79	23.76	1.80	126.9	45.36
KH-08	0.27	− 6.69	78.01	31.94	2.44	124.5	46.03
KH-09	− 9.40	− 9.37	39.86	296.54	0.13		
KH-10	0.00	− 5.60	51.18	39.35	1.30	124.5	40.50
KH-11	− 0.35	− 4.96	52.13	48.86	1.07	124.1	37.34
KH-12	− 0.39	− 7.25	38.83	36.37	1.07	122.9	48.85
KH-13	− 5.75	− 7.62	103.68	347.29	0.30	111.7	50.77
KH-14	− 2.27	− 4.62	161.32	191.86	0.84	120.3	35.69
KH-15	− 2.45	− 8.88	209.21	53.27	3.93	117.9	57.33
KH-16	− 0.10	− 6.10	206.08	69.46	2.97	124.1	43.04
KH-17	1.03	− 5.82	67.31	20.07	3.35	126.5	41.64
KH-18	0.75	− 4.11	28.84	58.52	0.49	126.8	33.19
KH-19	− 0.07	− 6.08	32.03	22.42	1.43	124.1	42.93
KH-20	0.01	− 5.89	111.13	35.16	3.16	124.4	41.96
KH-21	1.30	− 6.60	73.91	33.17	2.23	126.7	45.54
KH-22	1.26	− 6.00	34.67	35.07	0.99	126.9	42.52
KH-23	0.86	− 6.36	44.49	27.65	1.61	125.9	44.35
KH-24	1.01	− 7.25	57.93	19.59	2.96	125.8	48.85
KH-25	− 0.11	− 4.06	62.23	50.96	1.22	125.1	32.96
KH-26	1.10	− 4.84	194.63	234.54	0.83	127.1	36.74
KH-27	− 0.54	− 5.44	66.97	40.07	1.67	123.5	39.73
KH-28	− 0.57	− 6.66	40.65	27.81	1.46	122.8	45.88
KH-29	0.29	− 4.89	268.42	54.58	4.92	125.5	37.01

### Characteristics of carbon and oxygen isotope compositions

Carbon isotopes have strong stability and can better preserve the original sedimentary characteristics. Generally, after the formation of carbonate rocks, the impact of later transformation on them is relatively small. Normal marine carbonate rocks generally have δ^13^C values of 0 ~  ± 2‰^44,45^. However, oxygen isotopes are more sensitive to changes in the later stages of sedimentation. If oxygen isotope exchange occurs with atmospheric precipitation or hot underground fluids, the δ^18^O values decrease significantly^41,46^. In this study, two data points with poor reliability were removed, and the range of carbon and oxygen isotope changes in the Mesoproterozoic Jixian System was relatively narrow. The values of δ^13^C range from − 5.75 to 1.41‰, with an average of 0.16‰, and the values are mostly concentrated between − 1 and 1‰, while the values of δ^18^O range from − 8.88 to − 4.01‰, with an average of − 5.95‰, and the values are mostly concentrated from − 7 to − 4‰. The results are consistent with Hudson's^47^ distribution pattern of carbon and oxygen isotopes in marine carbonate rocks; this pattern is also consistent with the characteristics of carbon and oxygen isotope statistics in the region by Liu^48^ and Song^49^, which indirectly confirms that the region was in a long-term stable tidal flat environment during the Mesoproterozoic.

## Discussion

### Diagenetic environment

**Figure 4:**
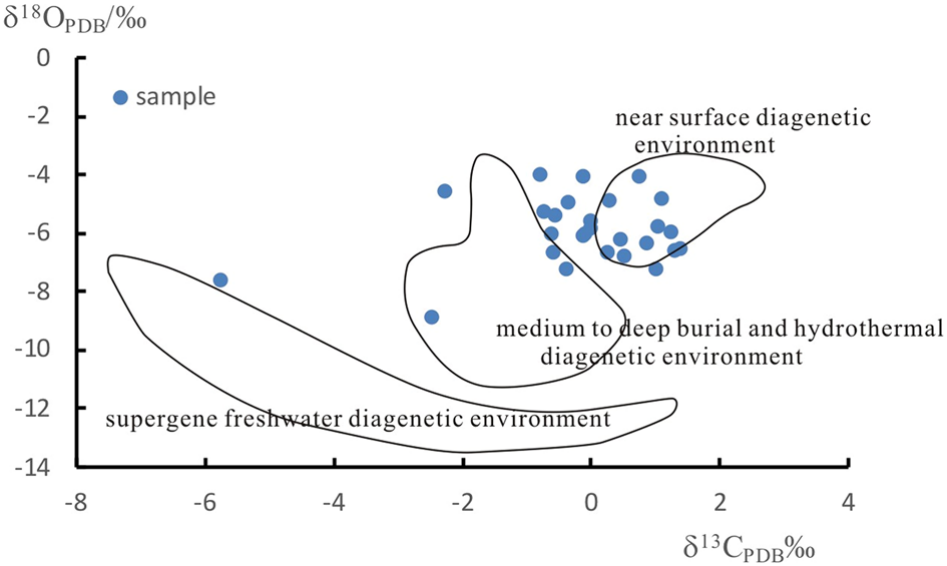
Intersection diagram of carbonate rock values of δ^13^C–δ^18^O^52,56^.

The diagenetic environment of carbonate rocks is the sum of various environmental factors, such as salinity, temperature, and redox properties^50^. Sea level affects diagenesis by controlling the movement state of underground fluid, thus determining the diagenetic environment and diagenetic process^51^. The environment determines the fabric of matter, and different diagenetic environments inevitably result in differences in carbon and oxygen isotope characteristics in their sediments^45^. Analyzing the diagenetic environment has become a key link in carbonate rock research, and identifying the diagenetic environment through the connection of carbon and oxygen isotopes has become an effective method^52,53^. Jiang^54^ divided the diagenetic environments of carbonate rocks into seawater environments, atmospheric freshwater environments, mixed water environments, burial environments, and epigenetic environments. Huang^55^ divided the diagenetic environments of carbonate rocks into early near-surface, epigenetic atmospheric freshwater, late mid-deep burial, and hydrothermal diagenetic environments. The carbon and oxygen isotopes of the carbonate rocks in this study mostly plot between the near-surface diagenetic environment and the medium–deep burial and thermal nocturnal diagenetic environments (Fig. 4), which indicates that the carbonate rocks in the study area were in a tidal flat sedimentary environment during the Mesoproterozoic era and later were buried to a certain depth; however, the burial depth was relatively shallow and the rocks were always in a near-surface environment, which is consistent with the results of no recrystallization of the carbonate rocks in the area. At the same time, due to the constraints of the shallow diagenetic burial environment on diagenesis, diagenesis has a weak degree of transformation of Mesoproterozoic carbonate rock samples, and it is possible to restore the paleosalinity and paleotemperature through carbon and oxygen isotopes.

### Paleosalinity (Z)

**Figure 5:**
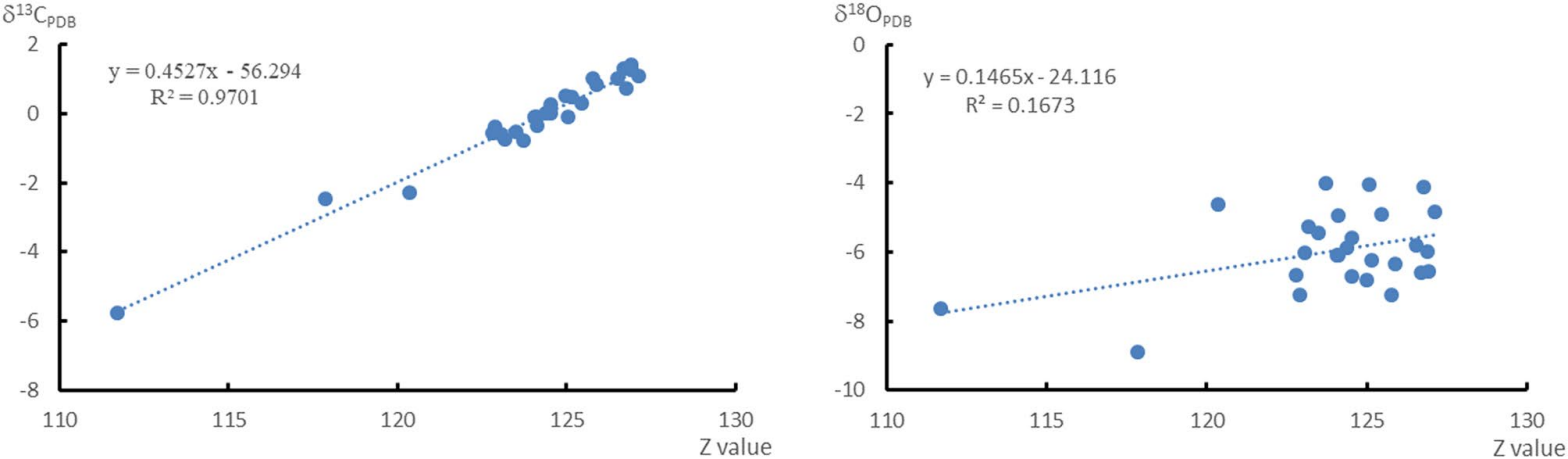
Relationship between carbon and oxygen isotopes and the Z value.

Generally, the oxygen isotope values in marine sediments increase with increasing paleosalinity^57^ because during the evaporation process, ^16^O is first carried by atmospheric precipitation, resulting in the enrichment of ^18^O in evaporated seawater^58^. Keith et al.^59^ proposed a classic salinity formula for distinguishing marine limestone from freshwater limestone during the Jurassic period and beyond based on the analysis and summary of a large amount of carbon and oxygen isotope data: Z = 2.048 × (δ^13^C_PDB_ + 50) + 0.498 × (δ^18^O_PDB_ + 50). It is believed that when the carbonate rock value of Z > 120, it formed by marine sedimentation, and when Z < 120, it formed by freshwater sedimentation. This formula has been widely used in restoring the paleosalinity of carbonate sedimentary environments in various geological historical periods^16,48,49^. In this study, most of the samples have Z values greater than 120, except for KH-13 and KH-15 (which have values less than 120 but near it), which indicates that the carbonate rocks in the region were formed in a stable marine environment; this is consistent with the research results of Liu et al.^56^ and also with the sedimentary environment of that period^29^. In terms of overall results, the range of Z value variation is not significant, indicating a relatively small climate change and stable tectonic environment during the Mesoproterozoic. However, further verification is needed to determine whether the Z value can serve as a quantitative indicator to characterize paleosalinity changes in the study area. Correlation coefficient analysis was conducted on carbon and oxygen isotopes and Z values, as shown in Fig. 5. The fitting equation between δ^13^C and Z values was y = 0.4527x − 56.294, with a correlation coefficient of 0.9701. The fitting equation between δ^18^O and Z values was y = 0.1465x − 24.116, with a correlation coefficient of 0.1673. The results indicate that the correlation between δ^18^O and Z values is strong, while the correlation coefficient between δ^13^C and Z values is weak, which may be closely related to the flourishing of algae and the rise and fall of sea level^14,16^.

### Paleotemperature (T)

During the formation of carbonate rocks, the main determining factor of the oxygen isotope content is temperature^60^. Although there are still many shortcomings in the method of quantitatively calculating the paleotemperature using oxygen isotopes, the changes in its content can qualitatively reflect the changes in paleotemperature in the diagenetic environment^57^. Previous researchers in the region used empirical formulas for trace elements Sr and T to determine that the paleotemperature of seawater during the deposition of carbonate rocks during the Mesoproterozoic was 31.1 °C^32,61^. However, as a sea-friendly element, Sr has strong migration ability and continues to be lost over time, resulting in a decrease in the Sr content in sediment^62^. Therefore, the temperature obtained using this method is not very accurate. Yu^63^ proposed a method for recovering the diagenetic temperature of carbonate rocks using oxygen isotopes: T = 13.85–4.54δ^18^O_PDB_ + 0.04(δ^18^O_PDB_)^2^. Using this method, it was found that the diagenetic temperature of the carbonate rocks in this study area was relatively concentrated, with an average of 42.32 °C, which is significantly different from the calculated T of trace element Sr isotopes. In addition, if the current surface temperature of the Ordos Basin is 20 °C and the normal geothermal gradient is 3 °C/100 m^64^, the burial depths of the Mesoproterozoic carbonate rocks in the study area were approximately 500–700 m, which is consistent with the analysis of the diagenetic environment mentioned earlier.

### Paleo-sea levels

Carbon isotopes have good indication significance. During diagenesis, the ^13^C composition of ancient carbonate rocks was very stable and almost unchanged, so the characteristics of the original sedimentary environment are well preserved^65^. Carbon in nature is composed of inorganic carbon and organic carbon. Inorganic carbon is relatively rich in the heavy isotope ^13^C, while organic carbon is relatively rich in the light isotope ^12^C. The relative composition of the two in the ocean determines the relative content of marine carbonate rocks and the composition of δ^13^C^66^. When the initial productivity of the ocean is high, the relative burial rate of organic carbon in seawater increases, and the relative increase in ^13^C in seawater leads to the formation of carbonate rocks with a positive δ^13^C excursion, while a warm climate, rising sea levels and thriving organisms all increase the relative burial rate of organic carbon in seawater, leading to a significant increase in seawater δ^13^C. Previous studies have shown that the evolution of the Proterozoic carbon isotope composition is mainly influenced by global sea level fluctuations^67^; therefore, the changes in δ^13^C can reflect the changes in the paleo-sea level during the sedimentary period.

The research area was located near the equator during the Mesoproterozoic era^56^, and the tidal flat environment during the deposition of carbonate rocks in the Ordos Basin had a high salinity and temperature. This may be due to its location near the equator, high temperature, relatively warm and humid climate, sustained thermal subsidence, and sea level rise under the expansion of the Qinqi Trough^29^, leading to an increase in seawater temperature. In addition, carbon isotope changes are generally closely related to biological productivity^16^. In this study, the characteristics of carbon and oxygen isotope changes are positively related. In combination with the shallow burial diagenetic environment, the Jixian System has undergone from weak diagenesis, and previous studies^68^ suggest that at relatively constant biological yields and higher temperatures, the isotope fractionation coefficient between carbonate rocks and seawater decreases, resulting in generally smaller carbon and oxygen isotope values and consistent carbon and oxygen isotope changes.

In summary, based on the analysis of carbon and oxygen isotope characteristics, paleosalinity, and paleotemperature, it is suggested that the environment and sea level of the Ordos Basin fluctuated during the middle Proterozoic, but the overall amplitude of various parameters changed relatively little, reflecting the stability of its tectonic environment and paleoclimate.

## Conclusion

1. The strata of the Mesoproterozoic Jixian System in the Ordos Basin were deposited in a near-surface environment without deep burial, and diagenesis was weak. The carbon and oxygen isotopes basically maintain the original geochemical characteristics.
2. In the carbonate rocks of the Mesoproterozoic Jixian System in the Ordos Basin, the ranges of δ^13^C_PDB_ and δ^18^O_PDB_ variation are relatively narrow, ranging from − 5.75 to 1.41‰ and − 8.88 to − 4.01‰, respectively, confirming that the study area was in a stable tidal flat sedimentary environment during the Mesoproterozoic. The paleosalinity Z values range from 111.7 to 127.1, and the paleotemperature values range from 32.71 to 57.33 °C, reflecting the warm paleoclimatic environment in the Mesoproterozoic in the study area.
3. The characteristics of changes in carbon and oxygen isotopes, paleosalinity (Z), paleotemperature (T), and a warm climate over a small range may be a good response to the location of the North China Block near the equator and its sustained thermal subsidence during the Mesoproterozoic.

## Data Availability

Data are available upon reasonable request; please contact the corresponding author.
